# Phosphoglycolate has profound metabolic effects but most likely no role in a metabolic DNA response in cancer cell lines

**DOI:** 10.1042/BCJ20180435

**Published:** 2019-02-19

**Authors:** Isabelle Gerin, Marina Bury, Francesca Baldin, Julie Graff, Emile Van Schaftingen, Guido T. Bommer

**Affiliations:** De Duve Institute and WELBIO, UCLouvain, Avenue Hippocrate 75, 1200 Bruxelles, Belgium

**Keywords:** DNA damage response, glycolysis, metabolic regulation

## Abstract

Repair of a certain type of oxidative DNA damage leads to the release of phosphoglycolate, which is an inhibitor of triose phosphate isomerase and is predicted to indirectly inhibit phosphoglycerate mutase activity. Thus, we hypothesized that phosphoglycolate might play a role in a metabolic DNA damage response. Here, we determined how phosphoglycolate is formed in cells, elucidated its effects on cellular metabolism and tested whether DNA damage repair might release sufficient phosphoglycolate to provoke metabolic effects. Phosphoglycolate concentrations were below 5 µM in wild-type U2OS and HCT116 cells and remained unchanged when we inactivated phosphoglycolate phosphatase (PGP), the enzyme that is believed to dephosphorylate phosphoglycolate. Treatment of PGP knockout cell lines with glycolate caused an up to 500-fold increase in phosphoglycolate concentrations, which resulted largely from a side activity of pyruvate kinase. This increase was much higher than in glycolate-treated wild-type cells and was accompanied by metabolite changes consistent with an inhibition of phosphoglycerate mutase, most likely due to the removal of the priming phosphorylation of this enzyme. Surprisingly, we found that phosphoglycolate also inhibits succinate dehydrogenase with a *K_i_* value of <10 µM. Thus, phosphoglycolate can lead to profound metabolic disturbances. In contrast, phosphoglycolate concentrations were not significantly changed when we treated PGP knockout cells with Bleomycin or ionizing radiation, which are known to lead to the release of phosphoglycolate by causing DNA damage. Thus, phosphoglycolate concentrations due to DNA damage are too low to cause major metabolic changes in HCT116 and U2OS cells.

## Introduction

Phosphoglycolate can be formed in all organisms during the repair of DNA damage. Oxidative stress or treatment with certain chemotherapeutic drugs (e.g. Bleomycin) and ionizing radiation leads to the oxidative cleavage of deoxyribose moieties, resulting in DNA 3′-ends that carry a phosphoglycolate group [1]. These 3′-phosphoglycolate groups can be removed either by releasing phosphoglycolate (via the enzyme APE1, [2–4]) or by releasing glycolate (via the enzyme TDP1, [5–7]) (see Supplementary Figure S1A). Phosphoglycolate is known to have an effect on two steps in glycolysis. On the one hand, it is an inhibitor of triose phosphate isomerase with a *K*_i_ value of ∼20 µM [8–10]. On the other hand, phosphoglycolate is expected to indirectly inhibit the activity of phosphoglycerate mutases, by stimulating the hydrolysis of the ‘priming phosphorylation’ of the enzyme [11,12]. This indicates that the link between phosphoglycolate release and metabolic changes could represent an ancient direct metabolic stress response, which might collaborate with more indirect effects of DNA damage on cellular metabolism [13,14] (Supplementary Figure S1A).

Two lines of evidence in the literature suggest a role of phosphoglycolate in the cellular response to oxidative stress. The first one is the observation that murine embryonic fibroblasts proliferate significantly slower upon inactivation of the enzyme phosphoglycolate phosphatase (PGP) [15], which is the enzyme that is believed to eliminate phosphoglycolate in cells. This difference was abolished under hypoxic conditions. Phosphoglycolate is expected to be formed by oxidative DNA damage and should accumulate to higher levels in PGP knockout cells than in wild-type cells. It was therefore tempting to speculate that increased concentrations of phosphoglycolate might play a role in the growth inhibition and some data were presented in support of this hypothesis [15] (see below).

The second line of evidence is the observation that reversible oxidation of PGP (also called AUM) can inhibit its activity and change its oligomerization state [16]. This suggests that under some conditions of oxidative stress, the elimination of phosphoglycolate by PGP might be compromised.

Taken together, oxidative stress might increase phosphoglycolate both at the level of its production (i.e. by promoting DNA damage) and at the level of its elimination (i.e. by inhibiting the enzyme PGP).

Unfortunately, the link between phosphoglycolate accumulation and the inhibition of cellular proliferation might not be quite as direct. When investigating the role of phosphoglycolate in the growth defect of PGP mutant MEFs, researchers used an inhibitor of the enzyme TDP1 to reduce phosphoglycolate levels. TDP1 is indeed one of the enzymes that clean up 3′-phosphoglycolate-DNA ends. Yet, this enzyme removes glycolate and not phosphoglycolate from DNA ends [5–7]. Therefore, inhibition of TDP1 is not expected to decrease phosphoglycolate concentrations but might rather increase phosphoglycolate concentrations since remaining 3′-phosphoglycolate-DNA ends could be processed by the enzyme APE1 (which actually leads to a release of phosphoglycolate) [3]. Thus, the observation that treatment with a TDP1 inhibitor rescues growth of PGP mutant MEFs does not support the notion that the growth inhibition is caused by phosphoglycolate but rather indicates that the cause for the growth inhibition in PGP mutant MEFs is likely more complex than previously assumed.

To make things even more complicated, the link between PGP and phosphoglycolate metabolism is less obvious than the name of this enzyme might suggest. Since its first description in 1977 [17], PGP has been described as a phosphatase that is most active on phosphoglycolate. While phosphoglycolate is abundant in plants, where it is formed as a side product of the enzyme Rubisco, in mammals, phosphoglycolate is not part of any known metabolic pathway but is primarily thought to be released during the repair of DNA damage.

Three lines of evidence suggest that the activity on phosphoglycolate might not be the most important function of PGP: first, we have previously shown that PGP plays a key role in cellular metabolism by eliminating two glycolytic side products, that otherwise interfere with glycolysis and the pentose phosphate pathway [18] (Supplementary Figure S1B). On the one hand, PGP eliminates 4-P-erythronate, which can be formed when the glycolytic enzyme glyceraldehyde-3-phosphate dehydrogenase (GAPDH) erroneously acts on erythrose 4-phosphate. On the other hand, PGP destroys also l-2-P-lactate, which can be formed when pyruvate kinase erroneously acts on l-lactate. Elimination of both metabolites is important since 4-P-erythronate is a potent inhibitor (*K_i_* < 1 µM) of the pentose phosphate pathway enzyme 6-P-gluconate dehydrogenase, and 2-P-lactate is a strong inhibitor of enzymes that synthesize the glycolytic activator fructose 2,6-bisphosphate. As expected, inactivation of PGP in cancer cells caused 10- to 50-fold increases in 4-P-erythronate and 2-P-lactate, as well as profound secondary disturbances including an inhibition of the glycolytic flux, a more than 100-fold increase in 6-P-gluconate concentration and reduced cell proliferation [18].

Second, it has been reported that a low activity of PGP on 3-P-glycerol is sufficient to modulate concentrations of this metabolite and might play a role in regulating cellular lipid metabolism [19,20].

Last but not least, while knockout or knockdown of PGP in mammalian cells has dramatic effects on 4-P-erythronate, 2-P-lactate and in some studies moderate effects on glycerol-3-phosphate [15,18–20], we were surprised not to detect any increase in phosphoglycolate beyond background levels in our previous study [18].

Here, we wanted to investigate how phosphoglycolate is formed in cells, what metabolic changes its accumulation can provoke and whether phosphoglycolate released during DNA repair might lead to noticeable metabolic changes.

## Experimental

### Lentiviral constructs

Lentiviral constructs driving expression of *Escherichia coli* gpmI were generated by inserting a PCR fragment (forward: ATA CAT AGC TAG CCA CCA TGT TGG TTT CTA AAA AAC CTA TG, reverse: TAT AAT GTA CAT TAT TCC ACG ATG AAC AGC) between the restriction sites NheI and BsrGI in the plasmid pOH425 [21]. The mouse Glyctk open reading frame was originally amplified from mouse liver cDNA and inserted into a prokaryotic expression vector. The open reading frame was then amplified by PCR and inserted into the plasmid pOH425 (details are available upon request). Inserts for the generation of lentiviral shRNA constructs were produced by amplifying synthetic oligonucleotides (IDT) (Supplementary Table S1) in a PCR with Phusion high-fidelity polymerase as described using primers TGA ACT CGA GAA GGT ATA TTG CTG TTG ACA GTG AGC G and TCT CGA ATT CTA GCC CCT TGA AGT CCG AGG CAG TAG GC [22]. Resulting PCR products were inserted via the restriction sites XhoI and EcoRI into an optimized miR-30 scaffold behind a turbo GFP expression cassette. This vector is similar to the constructs described by Fellmann et al. [22] but based on the vector pLVX-PURO (Clontech). Details about the construction of this vector are available upon request.

### Cell culture and lentiviral transduction

Cell lines were cultured in DMEM containing 4.5 g l^−1^ d-glucose, 10% foetal calf serum, 2 mM Ultraglutamine I (Lonza) and 100 U ml^−1^ Penicillin/Streptomycin (Lonza). PGP knockout cell lines were described previously [18]. Knockout cell lines in HCT116 cells (rescued or not with mouse PGP) were described previously [18]. The U2OS PGP knockout cell line was generated using the same approach as described previously [18]. To inactivate the PGP gene in polyclonal populations of the immortalized human fibroblast cell line HFF2-tert [23] (a generous gift of Anabelle Decottignies, UCLouvain, Belgium), we used the plasmid lentiCRISPR V2. Sequences of guide RNAs targeting human PGP or lacZ were inserted by ligating annealed oligonucleotides (see Supplementary Table S1) into the BsmBI site of this vector [24]. To generate recombinant lentiviruses (for overexpression of gpmI, knockdown of PKM/GLYCTK or lentiviral knockout of PGP), HEK293 T cells were transiently transfected with lentiviral vectors and second generation packaging plasmids psPAX2 and pMD2.G (kind gifts of Didier Trono, Addgene #12260 and #12259) using the calcium phosphate coprecipitation method as described previously [25,26]. Twenty-four hours after transfection, target cells were infected in the presence of 8 µg ml^−1^ polybrene (Sigma). Infected cells were selected for 4 days with 1.5 µg ml^−1^ of puromycin (ThermoFisher) and 300 µg ml^−1^ of hygromycin (Invivogen).

For the treatment with glycolate, glycolic acid (Sigma) was neutralized with sodium hydroxide and subsequently added to the medium at the indicated concentrations. Deuterated glycolate was synthesized by a reduction in glyoxylic acid with sodium borodeuteride. To this end, the two compounds were mixed at equimolar concentration and kept overnight at room temperature under basic pH. The mixture was neutralized with hydrochloric acid and stored at −20°C. A control solution was made by mixing glyoxylic acid and sodium borohydride to form non-labelled glycolate.

Before the induction of DNA damage, cells were plated at 400 000 and 300 000 cells per well of a six-well plate for HCT116 and U2OS cells, respectively, and let grow overnight. The following day, the medium was replaced by medium containing 10% (v/v) foetal bovine serum, 2 mM l-glutamine and 20 mM d-glucose. Cells were treated with 0, 5, 20 or 50 μM Bleomycin (Santa-Cruz, Heidelberg, Germany) or 5 Gy from a ^137^Cs source 24, 8, 4 and 0.5 h before harvesting the cells on the next day. As a positive control, where indicated, we added 5 mM glycolate 6 h before harvesting.

### Determination of the *K_i_* for succinate dehydrogenase and enolase

Succinate dehydrogenase (SDH) activity was assessed on rat liver mitochondria and on mitochondria obtained from PGP wild-type and knockout HCT116 cell lines. To this end, we isolated mitochondria as described previously [27] (after approval by the animal ethics committee of the medical faculty of the UCLouvain). Briefly, the liver from an overnight starved rat was homogenized in 10-fold excess of ice-cold isolation buffer (10 mM Tris buffer adjusted to pH 7.4 with MOPS, 1 mM EGTA, 200 mM sucrose) using three to four strokes with a Teflon-coated homogenizer operated at 1600 rpm. After centrifugation at 600 ***g*** at 4°C for 10 min, the supernatant was recovered. Mitochondria were collected from the supernatant by centrifugation at 7000 *g* at 4°C for 10 min and washed once in isolation buffer. For the determination of SDH activity in cell lines, cells were lysed in hypotonic phosphate buffer [20 mM KPi buffer (pH 7.4)] in three freeze–thaw cycles [28]. Protein concentration was determined using the bicinchoninic acid method (Thermo). Mitochondria were frozen down at high concentration (>80 µg ml^−1^) and adjusted to 5 µg ml^−1^ with isolation buffer on the day of the experiment.

SDH activity was determined using a published method [28]. Briefly, 10 µg of mitochondria were incubated at 37°C in a mixture containing 25 mM potassium phosphate buffer (pH 7.5), 1 g l^−1^ fatty acid-free BSA, 300 µM KCN, 0.015% (w/v) DCPIP (2,6-dichlorophenolindolphenol sodium salt dihydrate, Sigma) and the indicated concentrations of succinate and phosphoglycolate. The reaction was initiated by the addition of 50 µM decylubiquinone (DUB, Santa-Cruz) and followed by reading the absorption at 600 nm. Incubation with 10 mM malonate in control samples was used to determine non-specific background activity.

Enolase activity was determined in a coupled reaction containing pyruvate kinase and lactate dehydrogenase in a reaction mixture containing 20 mM HEPES (pH 7.5), 25 mM KCl, 1 mM DTT, 1 mM MgCl_2_, 1 mM ADP, 150 µM NADH using rabbit muscle enolase (Sigma, 3.5 mU ml^−1^), lactate dehydrogenase (Roche, 0.7 U ml^−1^) and pyruvate kinase (Roche, 1 U ml^−1^) as coupling enzymes. The reaction was initiated in the presence of the indicated concentrations of phosphoglycolate by the addition of the indicated concentrations of phosphoenolpyruvate (PEP) and followed by reading the absorption at 340 nm.

Kinetic parameters were determined by curve fitting using the Prism 7 software package (GraphPad) and are presented as mean ± s.e.m. of at least three independent determinations.

### Alamar Blue

Cell proliferation was assessed using the Alamar Blue assay [29] essentially as described previously [18]. Briefly, cells were plated in 96-well microtiter plates and cell viability was assessed 1 day and 4 days after plating by adding one-tenth volume of a 560 μM solution of resazurin (prepared in 0.9% NaCl solution from a 10-mM stock solution in DMSO), incubating for 4 h at 37°C and measuring fluorescence upon excitation at 540 nm and emission at 590 nm. To correct for differences in plating efficiencies, we normalized the results obtained after 96 h to the results obtained after 24 h.

### Western blot analysis

Cell lysates were prepared and western blots were performed using the same procedures as previously described [18]. Antibodies used were anti-PKM (Santa-Cruz, clone C-11, 1 : 1000), anti-PGP (Santa-Cruz, clone E10, 1 : 1000), anti-β-actin (Sigma, clone AC-15, 1 : 5000), anti-p53 (clone DO1, Santa-Cruz, 1 : 1000), anti-phospho-p53 (Cell Signalling, 1 : 1000), anti-P-H2AX (Millipore, 1 : 1000) and anti-tubulin (Sigma, 1 : 40 000).

### Analysis of metabolites by GC–MS

For metabolite analysis, cells were plated at 350 000 cells (HCT116), 125 000 cells (human fibroblasts) and 300 000 cells (U2OS) per well of a six-well plate. The following day, the medium was changed to DMEM without phenol red containing 3.6 g l^−1^ glucose, 10% dialysed foetal calf serum, 2 mM Ultraglutamine I (Lonza) and 100 U ml^−1^ Penicillin/Streptomycin (Lonza). Where indicated, phosphoglycolate was added at a concentration of 5 mM for the indicated times before harvesting. The extraction procedure of the intracellular metabolites, derivatization with MSTFA and methoxyamine and GC/MS analysis were performed exactly as described in Collard et al. [18]. Data were acquired in combined selected-ion monitoring (SIM) and scanning mode (68–700 *m/z*). Compounds were identified on the basis of their retention time and characteristic ions (Supplementary Table S2). SIM chromatograms of quantifier ions were integrated using Masshunter software (Agilent), and areas were normalized to total ion current [30]. GC/MS experiments were performed three or four times on separate days over a time frame of 6 months. In each experiment, three tissue-culture wells were quenched, extracted and derivatized separately for each condition. Each sample was then analysed separately (i.e. the samples were not pooled) by GC/MS. To facilitate the comparison between experiments performed on different days, values for each metabolite were normalized to the average of the values obtained in the untreated control cell line. In each experiment, the mean values for the normalized metabolite concentrations were calculated. The resulting three or four mean values (depending on the number of experiments) were then used to calculate means and s.e.m., which are presented in the figures. When multiple groups were compared, ANOVA was performed followed by a comparison of groups using Dunnett's test for multiple comparisons (when comparing to a single control group) or Tukey's test (when performing several pairwise comparisons). When the effects of PGP genotype and glycolate treatment were assessed, two-way ANOVA was performed, followed by post hoc tests corrected for multiple testing as described above. Absolute amounts of phosphoglycolate per milligram of cellular protein were estimated by linear regression of the peak areas extracted from SIM chromatograms from samples where known amounts of phosphoglycolate had been spiked in Ref. [18]. Absolute concentrations of phosphoglycolate were then estimated on the basis of a cellular water content of 4 μl per milligram protein [31].

For Figure 2A, isotopic distributions were corrected for the isotopic abundance using the known isotopic abundances of natural carbon and silicium with the Isocor package [32].

## Results

### Mammalian cells can produce phosphoglycolate by phosphorylation of glycolate mainly via pyruvate kinase

**Figure 1.**
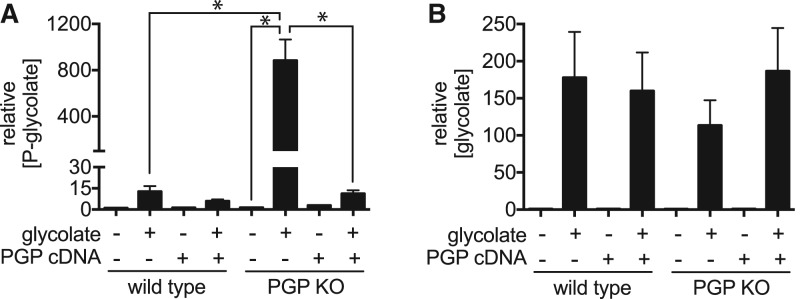
Glycolate supplementation leads to accumulation of phosphoglycolate in mammalian cells. Phosphoglycolate (**A**) and glycolate (**B**) levels were measured by GC/MS analysis in cell extracts 5 h after the addition of 5 mM glycolate to parental HCT116 cells and a PGP knockout clone, as well as to cells that have been complemented by transduction with a retroviral construct driving expression of a mouse PGP cDNA or an empty control construct [18]. Values are relative to the wild-type without glycolate addition and represent the mean ± s.e.m. of three independent experiments. Asterisks indicate *P* < 0.05 in post hoc *t*-test analysis corrected for multiple testing.

To investigate the cellular origin of phosphoglycolate, we first needed to identify a suitable experimental system. We turned to cell lines where the enzyme PGP had been genetically disabled. Previous work from our group has shown that this enzyme serves to eliminate two glycolytic side products, 2-P-lactate and 4-P-erythronate [18]. In these studies, we did not observe any increase in baseline phosphoglycolate levels in PGP knockout cell lines above 5 µM. This indicated that either very little phosphoglycolate is produced in these cell lines or that phosphatases other than PGP dephosphorylate and eliminate it. Here, we measured phosphoglycolate concentrations in PGP knockout cell lines derived from the HCT116 colorectal cancer and U2OS osteosarcoma cell lines, which were cultured either in the presence or absence of glycolate, a potential biosynthetic precursor of glycolate. In untreated cells, no significant difference in phosphoglycolate and 3-P-glycerol concentrations was observed between wild-type and knockout cells (Figure 1A,B, and Supplementary Figures S2C and 3A,B,H), whereas the other PGP substrates 4-P-erythronate and 2-P-lactate, as well as 6-P-gluconate, were strongly increased in knockout cell lines (Supplementary Figures 2A,B,F and S3C,E,F). Overall, these observations were similar to what we had previously observed in HCT116 cells [18]. In contrast, when we challenged wild-type and PGP knockout cell lines with 5 mM glycolate (i.e. a concentration in the range of those observed in patients with ethylene glycol intoxication, [33]), we observed a strong increase in phosphoglycolate levels (Figure 1A,B and Supplementary Figure S3A,B). Although glycolate concentrations were similar (Figure 1B and Supplementary Figure S3B), levels of phosphoglycolate in PGP knockout cell lines were consistently more than 5-fold higher than in wild-type cells (Figure 1A and Supplementary Figure S3A) and strongly reduced when PGP was re-expressed (Figure 1A), suggesting that PGP is the major enzyme responsible for the elimination of phosphoglycolate. Of note, while absolute concentrations of phosphoglycolate reached more than 1 mM in glycolate-treated PGP knockout HCT116 cells (Figure 1A), only a small reduction in viability was observed in these cell lines (Supplementary Figure S4)

**Figure 2.**
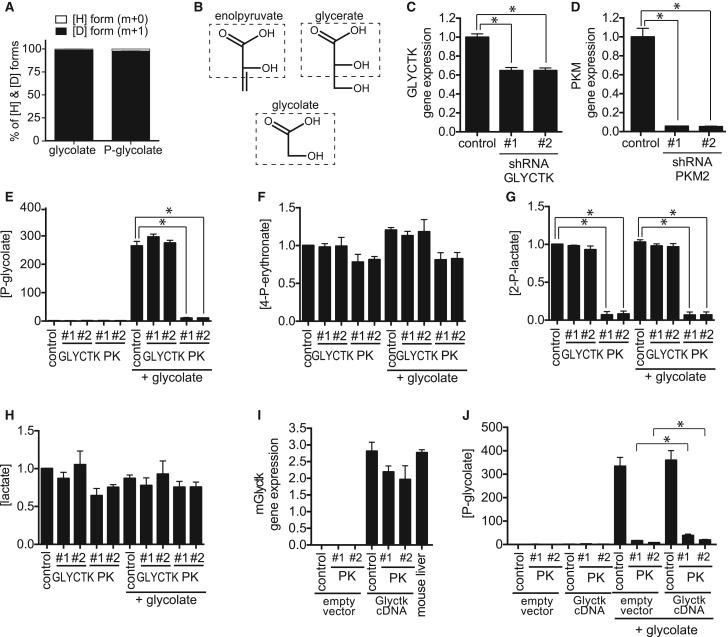
Phosphoglycolate is mainly formed by pyruvate kinase in the colorectal cancer cell line HCT116. (**A**) Relative abundance of glycolate and phosphoglycolate isotopomers was assessed by GC/MS in PGP knockout HCT116 cells (and wild-type cells, not shown) 6 h after treatment with 5 mM deuterated glycolate. Values shown represent the contribution of ^1^H and ^2^H after correction for the contribution of the trimethylsilyl part and the carbon backbone of glycolate. (**B**) Similarity between glycerate, glycolate and enolpyruvate. (**C**,**D**) Relative gene expression of GLYCTK (**C**) and PKM2 (**D**) in cells treated with two different shRNAs. (**E**–**H**) Relative concentrations of phosphoglycolate (**E**), 4-P-erythronate (**F**), 2-P-lactate (**G**) and lactate (**H**) were measured by GC/MS in GLYCTK and PKM2 knockdown cell lines 6 h after addition of 5 mM glycolate. Values represent the mean ± s.e.m. of four independent experiments and were normalized within each experiment to untreated wild-type cells. (**I**,**J**) We generated PGP knockout HCT116 cells where we knocked down PKM2 and overexpressed (or not) murine Glyctk mRNA. mRNA levels were assessed in these cell lines in comparison with mouse liver (**I**) and are represented normalized to the abundance of U6 mRNA (means ± s.e.m., *n* = 3). Phosphoglycolate concentrations (**J**) were assessed in these cell lines and are presented normalized to untreated control cells within each experiment (means ± s.e.m. of three independent experiments). Asterisks indicate *P* < 0.05 in post hoc *t*-test analysis corrected for multiple testing.

Phosphoglycolate could be formed either by direct phosphorylation of glycolate, or, possibly by a more complicated, as yet unknown, route involving its metabolism to glyoxylate. To distinguish between these possibilities, we treated cells with isotopically labelled glycolate carrying a deuterium on carbon 2. The only known metabolism of glycolate is indeed its conversion to glyoxylate, which leads to the removal of one of the two hydrogens that is directly bound to C2. Thus, if phosphoglycolate were formed after metabolization of glycolate to glyoxylate, we would expect a difference between the isotopomer distributions of phosphoglycolate and glycolate. In contrast, if phosphoglycolate were simply generated by phosphorylation of glycolate, we would expect similar isotopomer distributions for phosphoglycolate and glycolate. After treatment of cells, we extracted metabolites and analysed the mass isotopomer distributions of phosphoglycolate and glycolate by GC/MS. We observed that both were almost identical indicating that a kinase was likely responsible for the production of phosphoglycolate (Figure 2A).

Pursuing the question of which kinase could be involved we focused on pyruvate kinase (PKM2) and glycerate kinase (GLYCTK), which act on substrates that resemble glycolate (Figure 2B). Pyruvate kinase displays *in vitro* a side activity on glycolate that results in the formation of phosphoglycolate (and which is ∼10-fold higher than the side activity observed with l-lactate) [34]. While such activity has not been described for GLYCTK, this enzyme transfers a phosphate group to the hydroxyl attached to the alpha carbon of glycerate, comparable to the position of the phosphate group in phosphoglycolate [35].

To assess the contribution of these enzymes to the formation of phosphoglycolate, we transduced the HCT116 colorectal cancer cell line with lentiviral constructs driving the expression of shRNAs specific for PKM (coding for pyruvate kinases M1 and M2) or GLYCTK (coding for glycerate kinase). Quantitative RT-PCR analysis demonstrated a 40% and 85% decrease in transcript levels of GLYCTK and PKM, respectively (Figure 2C,D), and western blot analysis showed an ∼95% down-regulation of PKM protein in the cells expressing PKM shRNAs (not shown). Using GC/MS, we observed an 80–90% decrease in phosphoglycolate and 2-P-lactate levels when PKM was down-regulated, whereas these metabolites remained unchanged in cells in which GLYCTK gene was down-regulated (Figure 2E,G). Under these conditions, concentrations of the PGP substrate 4-P-erythronate were largely unaffected consistent with its production independent of pyruvate kinase (Figure 2F). Of note, these experiments were performed in the presence of pyruvate in the culture medium, to avoid major metabolic disturbances due to the knockdown of pyruvate kinase. Hence, lactate concentrations were not significantly reduced in PKM knockdown cells (Figure 2H), excluding that the reduction in 2-P-lactate would be caused by a reduction in lactate rather than by the down-regulation of pyruvate kinase.

Taken together, these results are consistent with the notion that the majority of phosphoglycolate and 2-P-lactate is produced by pyruvate kinase in this cell line. At this point, we could not exclude that glycerate kinase contributes for the formation of phosphoglycolate since we were not able to reduce GLYCTK transcript levels by more than 50%, despite attempting two different approaches (shRNA and lentiviral CRISPR/Cas9, data not shown). Given a more than 80% reduction in phosphoglycolate in cell lines where pyruvate kinase was knocked down, the contribution of GLYCTK in our experimental system can only be minor.

*In vivo*, GLYCTK expression is largely limited to the liver and the small intestine (GTEX portal, [36]), whereas expression in most cell lines is much lower. Thus, it could be conceivable that in these organs GLYCTK plays a major role in the formation of phosphoglycolate. To test this possibility, we overexpressed mouse Glyctk cDNA in cell lines where pyruvate kinase had been knocked down. As shown in Figure 2I, Glyctk mRNA levels upon overexpression were comparable to the ones observed in mouse liver. This led to a small increase in phosphoglycolate concentrations (Figure 2J), which represented less than 10% of the reduction that had been observed upon pyruvate kinase knockdown, consistent with the notion that GLYCTK can indeed contribute to a small extent to the production of phosphoglycolate.

Of note, one might ask the question whether phosphoglycerate kinase might also be involved in the production of phosphoglycolate. At first sight, this seemed very unlikely, since this kinase in its normal reaction phosphorylates the carboxylic function rather than a hydroxyl group. Nevertheless, we tested whether shRNA-mediated knockdown of PGK1 (the dominant form of PGK in the HCT116 cell line) might affect phosphoglycolate production. Introduction of two different shRNAs led to an 80–90% down-regulation of PGK1 mRNA levels (Supplementary Figure S5A). A concomitant increase in the ratio of triose phosphates to 3-phosphoglycerate concentrations indicated that the combined action of GAPDH and PGK1 was indeed inhibited (Supplementary Figure S5B). Yet, this did not lead to any reduction in cellular phosphoglycolate levels (Supplementary Figure S5C).

Taken together, we conclude that phosphoglycolate in HCT116 cells is mainly formed by the action of pyruvate kinase on glycolate.

### Accumulation of phosphoglycolate indirectly leads to an inhibition of phosphoglycerate mutase

**Figure 3.**
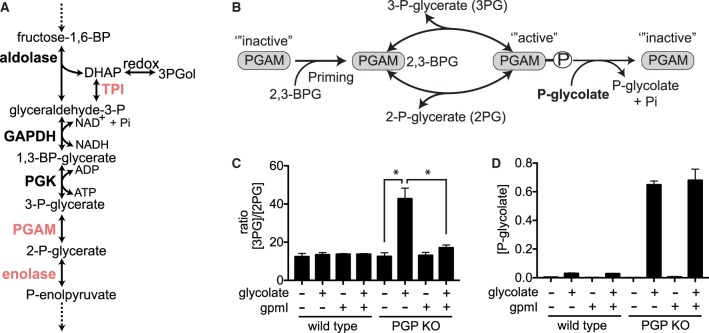
Phosphoglycolate accumulation blocks phosphoglycerate mutase — a process that can be prevented by expression of gpmI. (**A**,**B**) Schematic representations of part of glycolysis **(A)** and the reaction catalysed by phosphoglycerate mutase in the absence or presence of phosphoglycolate (**B**). (**C**,**D**) The ratios of 3-P-glycerate/2-P-glycerate concentrations (**C**) and phosphoglycolate concentrations (**D**) were assessed in parental HCT116 cells or in a PGP knockout clone expressing (or not) the 2,3-BPG-independent phosphoglycerate mutase, gpmI, from *Escherichia coli.* Values represent the mean ± s.e.m. of four independent experiments and are presented normalized to total ion current in arbitrary units. Asterisks indicate *P* < 0.05 in post hoc *t*-test analysis corrected for multiple testing.

Phosphoglycerate mutases (PGAMs) serve to interconvert 2-P-glycerate and 3-P-glycerate (Figure 3A). To catalyse this reaction, mammalian PGAMs need to be phosphorylated on a catalytic histidine residue in a reaction that uses 2,3-bisphosphoglycerate or (less efficiently) 1,3-bisphosphoglycerate (so-called priming phosphorylation) (Figure 3B) [37]. Under normal conditions, substrate binding leads to the formation of a 2,3-bisphosphoglycerate reaction intermediate and the release of either 2-P-glycerate or 3-P-glycerate, as well as a restoration of the enzyme-bound phosphate group. Of note, phosphoglycolate can also bind to mammalian PGAMs [38]. Yet, this does not lead to a 2,3-bisphosphoglycerate reaction intermediate, but rather to the hydrolysis of the phosphate group bound to the enzyme, thereby preventing the enzyme from performing its normal function [12]. Before the enzyme can work again, it therefore needs to be primed again.

To test whether phosphoglycolate concentrations in cells suffice to affect PGAM activity, we measured the ratio of 3-P-glycerate and 2-P-glycerate concentrations in PGP wild-type and knockout HCT116 cells (Figure 3C and Supplementary Figure S6A,B). This ratio increased more than 2-fold when we added glycolate to PGP knockout cells but remained unchanged in wild-type cells (Figure 3C). This increase was abolished upon re-expression of PGP (Supplementary Figure S2F). It was also observed in the PGP-deficient U2OS cells and, to a lower degree, in wild-type cells treated with glycolate (Supplementary Figure S7A–C) These observations were consistent with the hypothesis that high concentrations of phosphoglycolate observed in PGP knockout cells suffice to block PGAM activity by making it dependent on continuous re-priming. To test this hypothesis, we overexpressed gpmI, a 2,3-bisphosphoglycerate-independent phosphoglycerate mutase from *E. coli*. This enzyme does not require priming phosphorylation [39] and therefore should not be affected by the presence of phosphoglycolate. Indeed, when we treated PGP knockout cell lines overexpressing gpmI with glycolate, the increase in the ratio of 3-P-glycerate to 2-P-glycerate was significantly blunted (Figure 3C), whereas phosphoglycolate levels were unchanged (Figure 3D).

Surprisingly, PGP knockout cells incubated with glycolate showed lower PEP concentrations and a 2-fold increase in the ratio of 2-phosphoglycerate to PEP (Supplementary Figure S6D,E). In glycolysis, the reaction from 2-phosphoglycerate to PEP is catalysed by the enzyme enolase (Figure 3A). Our data therefore suggested that enolase might be inhibited by phosphoglycolate. Indeed, purified enolase was inhibited by 2-P-glycolate with a *K_i_* value of ∼160 µM (Supplementary Figure S6F).

### Metabolite changes are consistent with an inhibition of triose phosphate isomerase by phosphoglycolate in glycolate-treated PGP knockout cells

Phosphoglycolate is a well-known inhibitor (*K_i_* ≈ 20 µM) of the enzyme triose phosphate isomerase due to its similarity to a transition state occurring during the catalytic cycle of this enzyme [8–10]. If phosphoglycolate inhibits triose phosphate isomerase in cells, we expect to observe an increase in the ratio of dihydroxyacetone-P (DHAP, the substrate) to glyceraldehyde 3-P (GAP, the product) (see Figure 3A for schematic). Unfortunately, we were unable to assess glyceraldehyde 3-P levels reliably and therefore we cannot report this ratio. We did observe a substantial increase in the concentration of DHAP when glycolate was added to PGP knockout cells (Supplementary Figures S2D, S3G and S6C). This is consistent with the idea that phosphoglycolate accumulation indeed exerts an inhibition on triose phosphate isomerase in our experimental model. Yet, this conclusion has to be taken with caution since the increase in DHAP could also be caused by a build-up of metabolites upstream of phosphoglycerate mutase, which is inhibited by phosphoglycolate. The combined action of triose phosphate isomerase, glyceraldehyde-3-phosphate dehydrogenase and phosphoglycerate kinase is commonly assumed to be quite close to equilibrium [40]. Hence, the observed increases in 3-P-glycerate levels (Supplementary Figures S2E, S6A and S7B) are expected to lead to increases in upstream metabolites, including DHAP, even if triose phosphate isomerase is not inhibited. In the absence of a method that allows us to quantify glyceraldehyde-3-P and 1,3-bisphosphoglycerate, we cannot clearly distinguish between these two possibilities.

Of note, the analysis of triose phosphate activity in cellular lysates would not help us clarify this issue since the dilution of cellular lysates in the assay also dilutes the competitive inhibitor phosphoglycolate. Extrapolations to the situation in cells would therefore be difficult, in particular, if these measurements are performed with saturating substrate concentrations.

### Phosphoglycolate is a very efficient inhibitor of SDH

**Figure 4.**
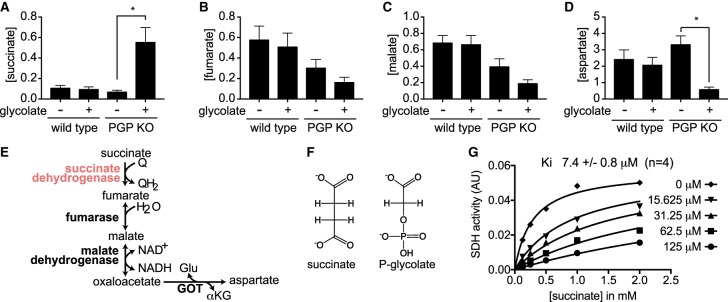
Phosphoglycolate blocks succinate dehydrogenase. (**A**–**D**) Concentrations of succinate (**A**), fumarate (**B**), malate (**C**) and aspartate (**D**) were determined by GC/MS analysis of wild-type or PGP knockout HCT116 cells in the presence or absence of 5 mM glycolate for 6 h. Values represent the mean ± s.e.m. of four independent experiments and are presented normalized to total ion current in arbitrary units. Asterisks indicate *P* < 0.05 in post hoc *t*-test analysis corrected for multiple testing. (**E**) Schematic representation of the metabolites measured in panels (**A**–**D**) in the citric acid cycle. (**F**) Structural similarity of phosphoglycolate and succinate. (**G**) succinate dehydrogenase (SDH) activity of rat liver mitochondria was assessed in the presence of varying concentrations of phosphoglycolate and succinate. The *K_i_* obtained from four independent experiments was 7.4 ± 0.8 µM.

When looking at the change in the concentrations of other metabolites, we noticed that the addition of glycolate to PGP knockout cells caused a marked increase in the concentration of succinate (Figure 4A), while it caused a decrease in the concentration of downstream metabolites in the Krebs cycle (fumarate and malate — Figure 4B,C). We also noted that aspartate, which is formed by transamination of oxaloacetate, was also markedly decreased (Figure 4D,E). These data suggested an inhibition of SDH by phosphoglycolate, most likely of the competitive type since phosphoglycolate is a structural analogue of succinate (Figure 4F). This was indeed confirmed by *in vitro* measurement of SDH activity in rat liver mitochondria; a competitive inhibition with a *K_i_* value of 7.4 µM was observed (Figure 4G). Of note, SDH activity in lysates from PGP wild-type and knockout cell lines was virtually identical (Supplementary Figure S8A) and inhibited by the addition of phosphoglycolate to a similar degree as in rat liver mitochondria (Supplementary Figure S8B). This is consistent with the idea that the inhibition of SDH by phosphoglycolate is responsible for the metabolite changes. At this point, it is unclear how phosphoglycolate enters the mitochondria, but, given the size and charge distribution of phosphoglycolate, the α-ketoglutarate/malate antiporter might be a candidate [41].

### Phosphoglycolate concentrations released during DNA damage repair are not sufficient to elicit metabolic changes in cancer cell lines

It has been previously reported that PGP knockout mice die during embryogenesis [15]. Embryonic fibroblasts obtained from PGP knockout embryos grow much slower than their wild-type counterparts under normoxia but grow normally under hypoxia. This profound oxygen-dependent growth inhibition has been attributed to the accumulation of oxidative DNA damage lesions, which can lead to the formation of phosphoglycolate [15]. Yet, it was unclear whether the phosphoglycolate produced from the repair of 3′-phosphoglycolate ends (Supplementary Figure S1A) would be sufficient to modulate cellular phosphoglycolate concentrations to a relevant extent.

**Figure 5.**
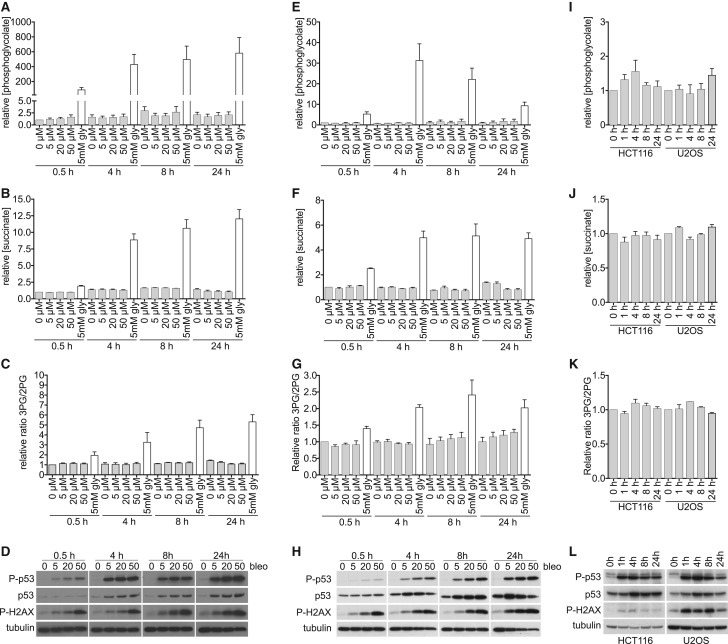
Phosphoglycolate concentrations observed upon DNA damage with Bleomycin are far from the concentrations that lead to metabolic effects. (**A**–**H**) PGP knockout clones derived from the cell line HCT116 (**A**–**D**) and U2OS (**E**–**H**) were incubated for the indicated times with the indicated concentrations of Bleomycin, grey columns or 5 mM glycolate, white columns. Using GC/MS, we analysed phosphoglycolate concentrations (**A**,**E**), succinate concentrations (as a measure of the inhibition of succinate dehydrogenase) (**B**,**F**) and the ratio of 3-P-glycerate/2-P-glycerate (as a measure of the inhibition of PGAM) (**C**,**G**). The cellular DNA damage response was assessed by western blot analysis using p53, phospho-p53 and phospho-H2AX antibodies. Equal loading was verified by hybridization with a β-tubulin antibody (**D**,**H**). Values represent the mean ± s.e.m. of three independent experiments and are normalized to the wild-type untreated cells at 0.5 h. (**I**–**L**) metabolite concentrations (**I**–**K**) and DNA damage markers (**L**) were assessed in PGP knockout clones obtained from the indicated cell lines at the indicated time points after 5 Gy irradiation. Data are presented as described in (**A**–**H**).

To test this, we treated PGP knockout lines obtained from the HCT116 colorectal carcinoma and U2OS osteosarcoma cell line with Bleomycin, the chemotherapeutic agent that is best characterized to lead to the production of phosphoglycolate [42], and with ionizing radiation (Figure 5). Cells indeed underwent considerable DNA damage as documented by the western blots for DNA damage response pathway components p53, phospho-p53 and phospho-H2AX (Figure 5D,H,L). Phosphoglycolate levels, as well as surrogate markers indicative of an inhibition of SDH or PGAM, were assessed by GC/MS. Yet, we did not observe any significant increase in phosphoglycolate upon treatment with Bleomycin or irradiation (Figure 5A,E,I). Likewise, we did not observe any change in succinate or 3PG/2PG ratios, indicating that no inhibition of SDH or phosphoglycerate mutases had occurred (Figure 5B,C,F,G,J,K). This demonstrates that phosphoglycolate production during DNA damage repair is too small to lead to major metabolic changes in our experimental systems.

## Discussion

Phosphoglycolate is a common metabolite in photosynthetic plants, where it is formed when Rubisco erroneously uses O_2_ instead of CO_2_ as a substrate [43]. In contrast, in mammals, phosphoglycolate is not a part of any known metabolic pathway. In the present study, we elucidated the formation and biological effects of phosphoglycolate in mammalian cell lines.

At baseline, phosphoglycolate levels were <5 µM in both wild-type and PGP knockout cell lines. In contrast, we observed a strong increase when cells were treated with exogenous glycolate. This increase was more than 5-fold stronger in PGP knockout cell lines consistent with the idea that PGP is the main enzyme responsible for the degradation of this metabolite. Using isotopically labelled glycolate, we demonstrated that phosphoglycolate was mainly synthesized by direct phosphorylation. Two kinases are known to potentially phosphorylate a hydroxyl group present on carbon 2 of a carboxylic acid. One is pyruvate kinase, which is known to display side activities that lead to the phosphorylation of lactate and glycolate [44]. Another one is glycerate kinase, which converts glycerate to 2-P-glycerate in vertebrates and in bacteria [45]. Knockdown of pyruvate kinase M1/2 led to a more than 80% decrease in the formation of phosphoglycolate in PGP-deficient cells, while this was not the case if the glycerate kinase gene was inactivated. These findings indicate that the side activity of pyruvate kinase is responsible for the formation of phosphoglycolate.

Two additional observations are important in these experiments. First of all, we also demonstrated that another PGP substrate, 2-P-lactate, is also produced in a pyruvate kinase-dependent manner (whereas the PGP substrate 4-P-erythronate is largely unaffected). This means that PGP, in fact, eliminates two side products of pyruvate kinase, phosphoglycolate and 2-P-lactate, and a side product of glyceraldehyde 3-phosphate dehydrogenase, 4-P-erythronate [18]. It is remarkable that the rather broad substrate spectrum of PGP (i.e. a certain lack of specificity) allows it to correct the errors committed by two major glycolytic enzymes. Secondly, treatment of cells with 5 mM glycolate at first sight seems like something extremely non-physiological. Yet, it should be noted that intoxications with ethylene glycol commonly lead to similar concentrations in blood [33]. As such, the metabolic changes observed in glycolate-treated PGP knockout cells indeed represent the situation that cells would encounter during ethylene glycol intoxication in the absence of PGP. Obviously, this situation is exceedingly rare, and there need to be other reasons why PGP has retained its activity towards phosphoglycolate during evolution. One explanation would be that the requirement to maintain activity on both 2-P-lactate and 4-P-erythronate inevitably leads to some activity on phosphoglycolate. Retaining the activity towards phosphoglycolate, which is almost absent in cells, might simply not have been a disadvantage. Of course, it is also conceivable that the dephosphorylation of phosphoglycolate might have represented a significant selective advantage at some point during evolution. For example, inactivation of the yeast homologue of PGP, pho13, leads to significant increases in phosphoglycolate levels [18]. At this point, it is unclear why this would represent a selective advantage. Yet, we did not further pursue this since preliminary experiments indicated that phosphoglycolate production in yeast might be more complex than in mammals (data not shown).

In the absence of glycolate addition, the level of phosphoglycolate is below 5 µM, even in cells that are PGP-deficient. This is most likely due to the fact that glycolate levels are very low. In mammalian cells, glycolate essentially results from the reduction in glyoxylate by hydroxypyruvate/glyoxylate reductase, an enzyme present in the cytosol and in mitochondria [46]. This reaction prevents glyoxylate from being irreversibly converted to oxalate by lactate dehydrogenase in the cytoplasm [46,47]. Glycolate is converted back to glyoxylate and eventually glycine in the peroxisomes thanks to the action of glycolate oxidase (l-2-hydroxyacid oxidase I) and alanine glyoxylate aminotransferase (AGT) [48,49]. This system appears to be very efficient in the cell models that we used and thereby prevents the formation of phosphoglycolate.

These observations left open the possibility that DNA damage could be a relevant source of phosphoglycolate, which could lead to metabolic effects with an important role in the cellular DNA damage response. To test this, we first investigated secondary metabolic changes under conditions where phosphoglycolate concentrations were moderately (i.e. glycolate-treated wild-type cells) or highly (i.e. glycolate-treated PGP knockout cells) elevated. We observed that high concentrations of phosphoglycolate lead to profound metabolic disturbances. Some of these changes were expected based on the literature. Yet, others (such as the inhibition of SDH) are described here for the first time.

On the one hand, we observed metabolite changes that are consistent with an inhibition of phosphoglycerate mutase (Supplementary Figure S1C). This result was expected since phosphoglycolate is well known to cause the dephosphorylation of a catalytic histidine residue in phosphoglycerate mutases [38]. To be able to act, phosphoglycerate mutases then need to be phosphorylated by 2,3-bisphosphoglycerate (or less efficiently 1,3-bisphosphoglycerate) (Figure 3B). In line with this model, we were able to prevent the inhibition of the phosphoglycerate mutase step by expressing a bacterial phosphoglycerate mutase enzyme that does not use a phosphohistidine intermediate [45] (Figure 3C).

On the other hand, we made the surprising observation that very high concentrations of phosphoglycolate were associated with a strong increase in succinate concentrations (Figure 4A). *In vitro* studies using purified mitochondrial extracts subsequently allowed us to establish that phosphoglycolate competitively inhibits SDH with a *K_i_* value of <10 µM. This competition is likely a consequence of a certain structural similarity and similar charge distribution between 2-P-glycolate and succinate (Figure 4F).

These striking metabolic changes were observed when we treated PGP knockout cell lines with glycolate, which led to very high levels of phosphoglycolate. In contrast, secondary effects were almost absent when we treated wild-type cells with glycolate, even though this led to clear increases in phosphoglycolate levels reaching more than 40 µM. With regard to the changes of glycolytic intermediates, this is not surprising, since the enzymes inhibited by phosphoglycolate (TPI, PGAM and enolase) are commonly believed to act close to equilibrium and have a large reserve capacity [40]. Thus, inhibition of these enzymes is not necessarily expected to lead to changes in metabolite concentrations. In fact, even a strong change in the ratio of substrate to product concentrations should not be taken as an indication that glycolytic flux is changed. Yet, this does not mean that inhibition of these enzymes is always irrelevant. For examples, inhibition of PGAM activity and the resulting increase in 3-P-glycerate concentrations can lead to an increase in serine synthesis [37,50]. With regard to the inhibition of SDH, we also need to take into account that phosphoglycolate first needs to be taken up in the mitochondria and that effective concentrations there might be significantly lower than in the cytoplasm.

We reasoned that DNA damage-induced phosphoglycolate release could only lead to secondary metabolic changes if it actually increased cellular phosphoglycolate concentrations. To test this, we treated PGP knockout cells with Bleomycin, the prototypical chemotherapeutic drug that is known to lead to phosphoglycolate release, and with ionizing radiation. In two different cell lines (HCT116 and U2OS), we did not find any significant increase in Bleomycin-treated or irradiated PGP-deficient cells. This indicated that it is extremely unlikely that phosphoglycolate released during DNA damage could directly contribute to secondary metabolic changes in our experimental systems.

The overall number of oxidative hits in human fibroblasts has been estimated to be between 9000 and 31 500 per day [51,52] and the majority lead to the formation of 8-oxoguanine. Even if we make the unrealistic assumption that all lesions would result in phosphoglycolate release and if we consider very small cell types like mouse lymphocytes with a cellular volume of 120 fl [53], this only amounts to an increase in 0.4 µmol l^−1^ per day, which, in 1 day, would lead to a concentration that is at least 10-fold lower than the *K_i_* value for any of the known inhibitory effects of phosphoglycolate. Thus, it is unlikely that under normoxic conditions, proliferating cell types can accumulate sufficient phosphoglycolate to account for measurable metabolic effects.

Yet, it has previously been suggested that DNA damage-dependent phosphoglycolate played a role in the growth defect in PGP knockout MEFs [15]. Segerer et al. observed that these cells grow dramatically slower than their wild-type counterpart. Interestingly, this difference was abolished when these cells were cultured under hypoxic conditions or when they were treated with an inhibitor of TDP1 [19], one of the enzymes that is responsible for the repair of 3′-phosphoglycolate ends (Supplementary Figure S1A). At first sight, this might suggest that oxygen-dependent phosphoglycolate release might play a role in the growth deficit of MEFs. Yet, this conclusion is incorrect, since TDP1 removes glycolate and not phosphoglycolate from DNA ends [5–7] (see schematic in Supplementary Figure S1A). Therefore, inhibition of TDP1 is not expected to decrease phosphoglycolate concentrations but might rather increase phosphoglycolate formation since remaining 3′-phosphoglycolate-DNA ends could be repaired by the enzyme APE1 (which actually leads to a release of phosphoglycolate) [3]. Thus, the observation that treatment with a TDP1 inhibitor rescues growth of PGP mutant MEFs does not allow any conclusion on the involvement of phosphoglycolate in this process but rather indicates that the cause for the growth inhibition in PGP mutant MEFs is likely more complex than previously assumed.

Of note, we do not encounter a major reduction in viability when we genetically inactivate PGP in human normal fibroblasts (Supplementary Figure S9). Comparable to our observations in cancer cell lines, human fibroblasts with complete PGP knockout show 25-fold, 9-fold and 110-fold increases in 2-P-lactate, 4-P-erythronate and 6-P-gluconate, respectively (Supplementary Figure S9B,C,F), whereas phosphoglycolate and 3-P-glycerol levels remain unchanged or are reduced (Supplementary Figure S9D,E). This suggests that the difference in behaviour in MEFs is not just a question of being ‘normal’ versus being ‘cancerous’. MEFs are known to be exquisitely sensitive to oxidative stress and show more oxidative DNA damage than their human counterparts [54]. Thus, it might be conceivable that MEFs for unknown reasons show extraordinarily high oxygen-dependent phosphoglycolate release or changes in 3-P-glycerol metabolism. Alternatively, PGP knockout MEFs might simply not be able to maintain sufficient NADPH synthesis and anti-oxidative defense, if 4-P-erythronate accumulates and inhibits 6-P-gluconate dehydrogenase activity similar to the situation in other PGP knockout cells (see Supplementary Figures S1B, S3F and S9F). Future experiments in MEFs will have to resolve these questions.

The situation might be quite different when cells are treated with Bleomycin or ionizing radiation, since in cell-free conditions, these treatments can lead to very high concentrations of 3′-phosphoglycolate ends [55]. Yet, in our experiments, we did not observe any significant increase in phosphoglycolate concentrations upon DNA damage in two different cell lines, although several different concentrations and time points were assessed. This indicates that in HCT116 and U2OS cells, phosphoglycolate does not play a role in a metabolic response to DNA damage.

## Abbreviations

GAPDH: glyceraldehyde-3-phosphate dehydrogenase
PEP: phosphoenolpyruvate
PGP: phosphoglycolate phosphatase
SDH: succinate dehydrogenase
SIM: selected-ion monitoring
