# Supplementary material for: Phosphoglycolate has profound metabolic effects but most likely no role in a metabolic DNA response in cancer cell lines
Source: Biochem J. 2019 Feb 19;476(4):629–43. doi: 10.1042/BCJ20180435 (PMC6380167; doi:10.1042/BCJ20180435)
Supplement: Supplementary Figures and Tables [file BCJ-476-629-s1.pdf]

SUPPLEMENTARY DATA

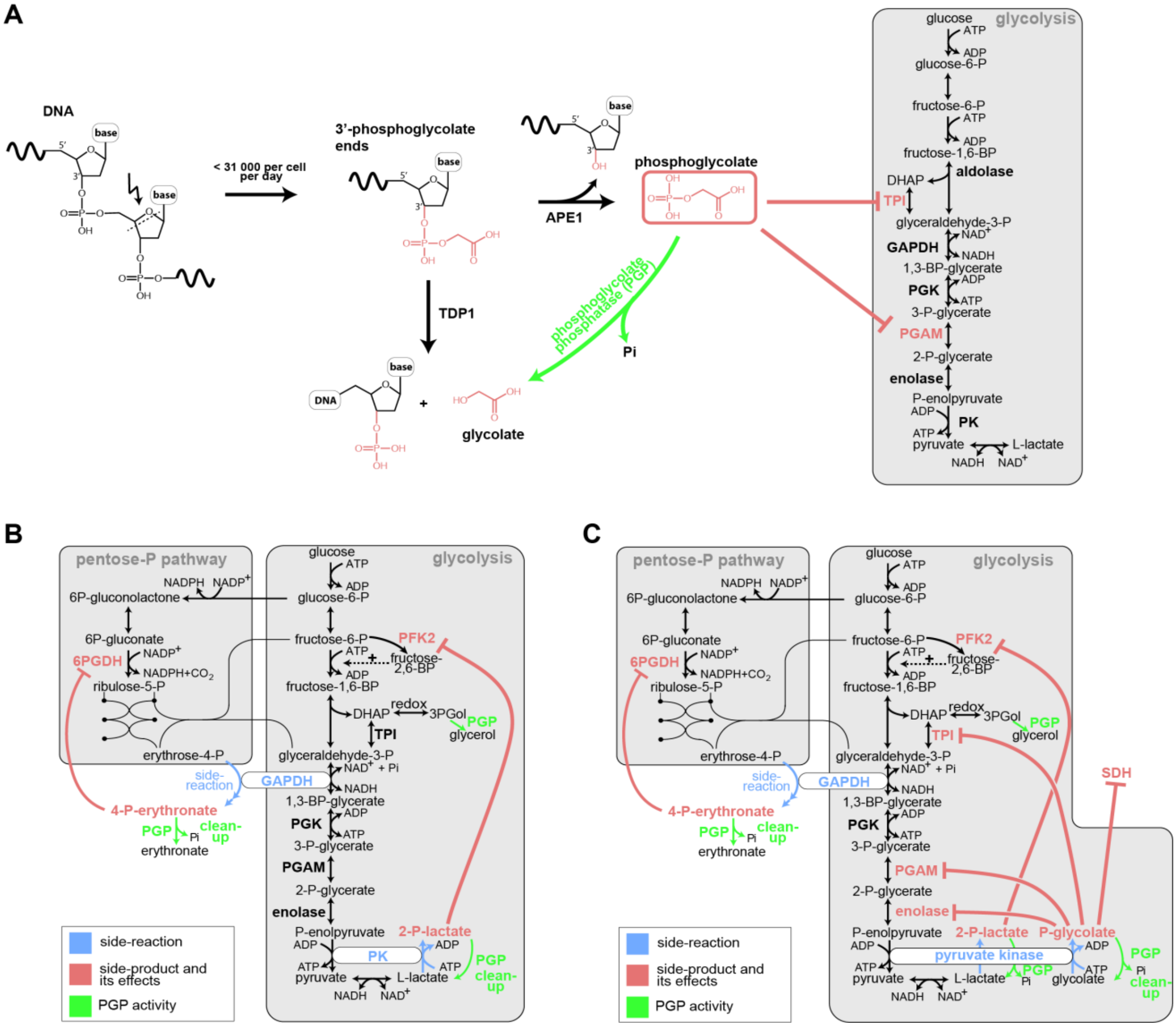

Supplementary Figure 1: Schematic representation of the pathways under investigation

A, <u>Schematic representation of the link between DNA damage, phosphoglycolate and potential metabolic changes</u>. Cleavage of the deoxyribose backbone can lead to the formation of 3' ends carrying phosphoglycolate groups. These 3'-phosphoglycolate ends are repaired either by removal of phosphoglycolate by the enzyme APE1 [1, 2] or by removal of glycolate by the enzyme TDP1 [3-5].
Phosphoglycolate is expected to be dephosphorylated by the enzyme phosphoglycolate phosphatase. Accumulation of phosphoglycolate could lead to an inhibition of triose phosphate isomerase (TPI) and, indirectly, of phosphoglycerate mutase (PGAM).

B, <u>Schematic representation of a major metabolic clean-up function of the enzyme phosphoglycolate phosphatase revealed in previous work</u> [6]. When the enzymes pyruvate kinase and glyceraldehyde 3-P dehydrogenase erroneously act on L-lactate and erythrose 4-P, they form 2-P-L-lactate and (indirectly) 4-P-erythronate, respectively. In normal cells, these side-products are rapidly dephosphorylated by the enzyme phosphoglycolate phosphatase. Yet, when this enzyme is absent, we observed a strong increase in these metabolic side products. We also observed more than 50-fold increases in 6-P-gluconate concentrations, most likely due a very efficient inhibition of the enzyme 6-P-gluconate dehydrogenase (6PGDH) by 4-P-erythronate. Furthermore, we observed a reduction of glycolytic flux, which is most likely at least in part caused by the inhibition of phosphofructokinase-2 by L-2-P-lactate [6].

In addition, several papers described increases of 3-P-glycerol (3PGol) when PGP is knocked down or knocked out [7-9]. Yet, it should be noted that the catalytic efficiency for this metabolite is ≈ 100x lower than the one for phosphoglycolate, 2-P-L-lactate and 4-P-erythronate[6].

C, Schematic representation of biogenesis and metabolic effects of phosphoglycolate observed in the present work.

Phosphoglycolate in our experimental system is largely synthesized by phosphorylation of glycolate. High concentrations of phosphoglycolate can lead to an inhibition of triose phosphate isomerase, phosphoglycerate mutase, enolase and succinate dehydrogenase (SDH).

BP, bisphosphate; P, phosphate; 6PGDH, 6-P-gluconate dehydrogenase; PFK2, phosphofructokinase 2; 3PGol, 3-P-glycerol; P-glycolate, phosphoglycolate; DHAP, dihydroxyacetone phosphate; PGK, phosphoglycerate kinase; PGP, phosphoglycolate phosphatase; TPI, triose phosphate isomerase; PGAM, phosphoglycerate mutase; PK, pyruvate kinase; SDH, succinate dehydrogenase. Not all cofactors are listed.

The word “redox” in the reaction between dihydroxyacetone phosphate (DHAP) and 3-P-glycerol (3PGol) indicates that this reaction is determined both by the cytoplasmic $NADH/NAD^+$ ratio and the ratio of reduced to oxidized coenzyme Q.

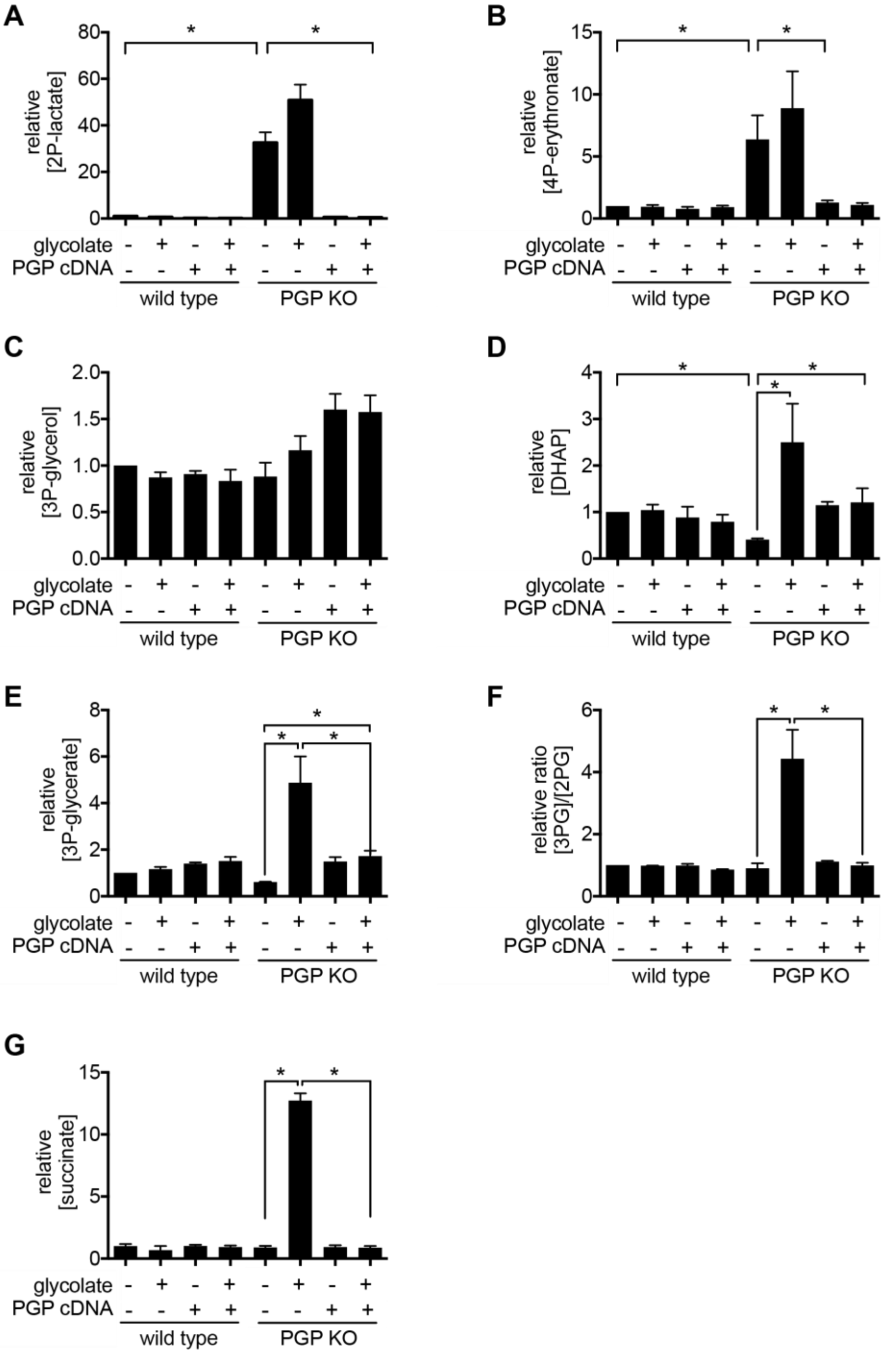

Supplementary Figure 2. Relative metabolite content of cells described in Figure 1. Concentrations have been assessed by GC/MS in parental HCT116 cells or in a PGP knockout clone after treatment or not with glycolate. Cell lines were either transduced with a retroviral construct driving expression of mouse PGP cDNA ("+") or an empty control construct ("-"). Values represent the mean ± s.e.m. of three independent experiments and are presented relative to the untreated wild type cells.

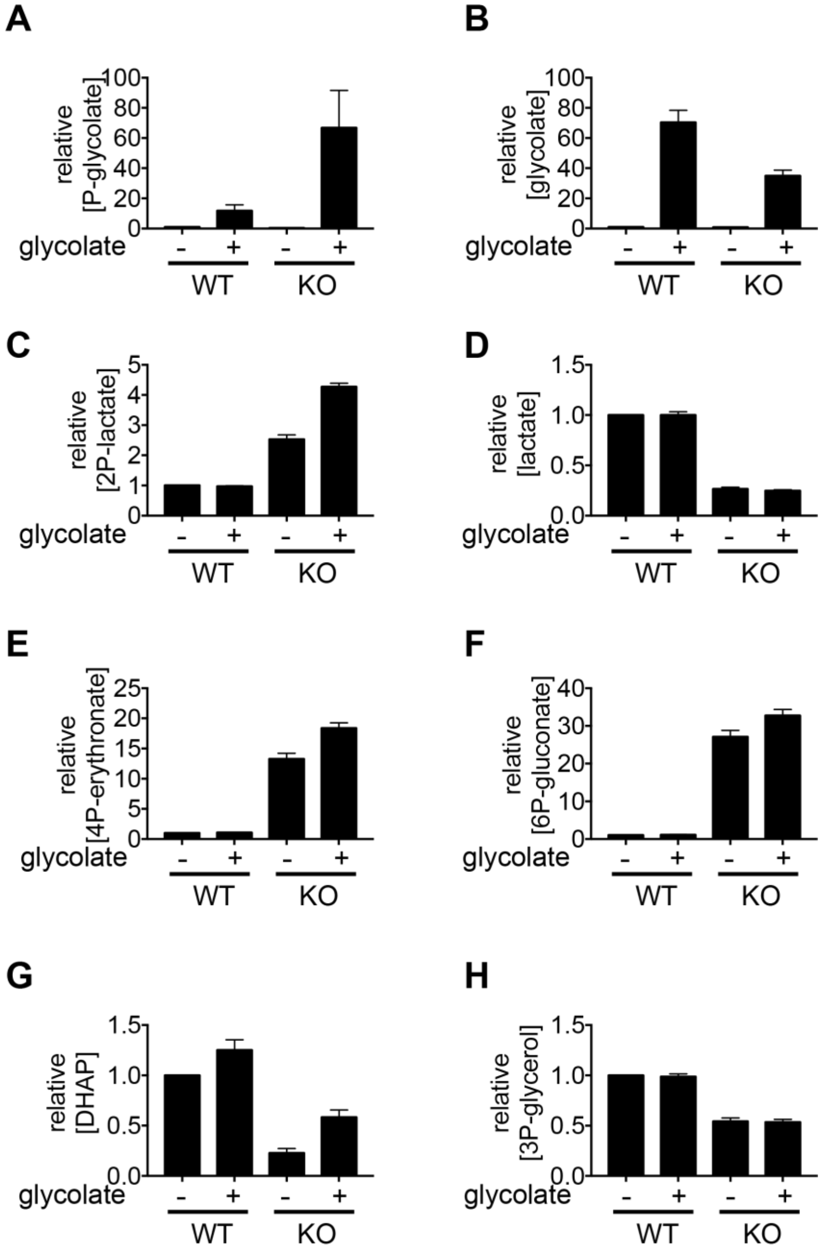

Supplementary Figure 3. Relative metabolite content of wild type and PGP knockout U2OS cells in the presence and absence of glycolate treatment.

Concentrations of phosphoglycolate (A), glycolate (B), 2-P-lactate (C), lactate (D), 4-P-erythronate (E), 6-P-gluconate (F), dihydroxyacetone phosphate (DHAP) (G) and 3-P-glycerol (H) were measured by GC-MS and were normalized to the untreated wild type cells within each experiment. Values represent means ± s.e.m. of four independent experiments and are presented relative to the untreated wild-type cells.

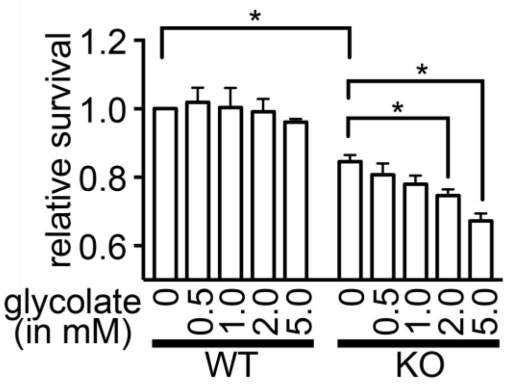

Supplementary Figure 4: Survival of wild type and PGP knockout cells in the presence of glycolate.
HCT116 wild type and PGP knockout cells were plated in the presence of the indicated concentrations of glycolate. Viability was assessed with the Alamar blue assay 1 and 4 days after plating. The signal obtained at day 4 was normalized to the one obtained at day 1 to correct for differences in plating. Values are means +/- s.e.m. of four independent experiments performed in 6 replicates, and are presented relative to the untreated wild type cells.

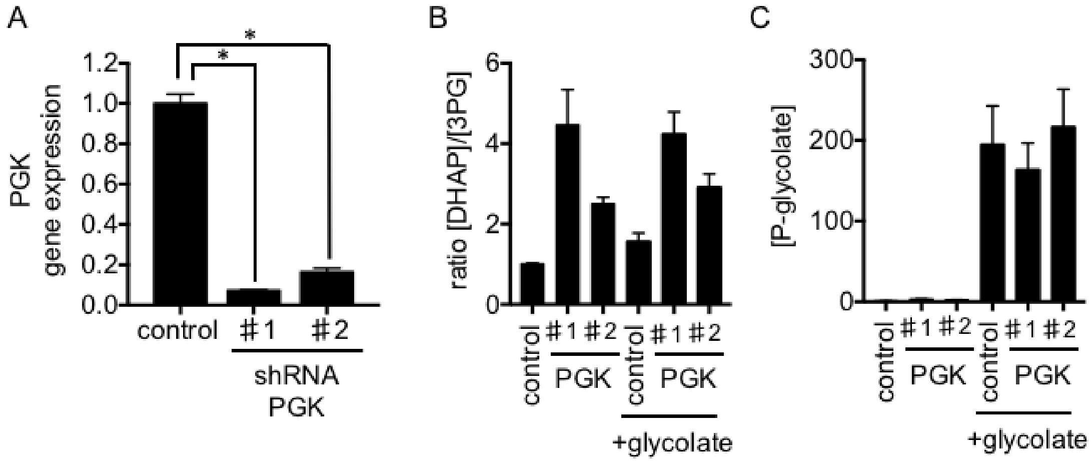

Supplementary Figure 5. Phosphoglycerate kinase does not contribute to the formation of phosphoglycolate.

A, Phosphoglycerate kinase 1 mRNA levels (relative to U6) were assessed by qPCR in HCT116 PGP knockout clone C4 expressing a non-silencing control shRNA or two independent shRNAs targeting human PGK1. Values represent means and s.e.m. from three independent experiments. **B&C,** Dihydroxyacetone phosphate (DHAP), 3-P-glycerate (3PG) (expressed as concentration ratios in B) and phosphoglycolate concentrations (C) were determined by GC-MS in the cell lines described in A after incubation or not with glycolate. Values were normalized to those obtained in untreated control shRNA expressing cells in each experiments and represent the means +/- s.e.m. of three independent experiments.

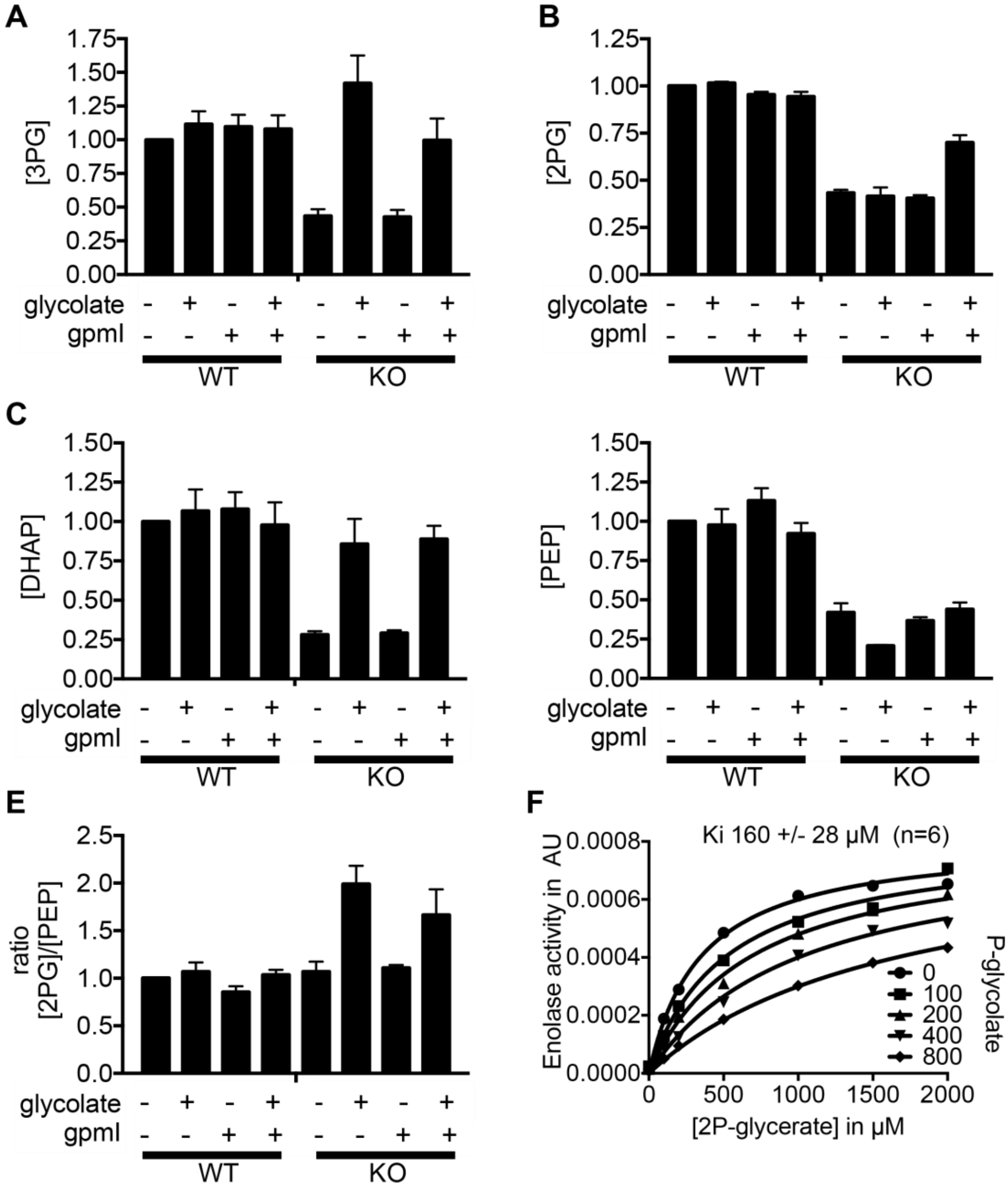

Supplementary Figure 6. Relative metabolite content of cells described in Figure 3.

A-E Concentrations are relative to the untreated wild type cells and have been assessed in parental HCT116 cells or in a PGP knockout clone expressing (or not) the 2,3-BPG-independent phosphoglycerate mutase gpmI from *Escherichia coli.* Values represent the means ± s.e.m. of four independent experiments and are presented relative to the untreated wild-type cells. **F,** Enolase activity was assessed in the presence of the indicated concentrations of phosphoglycolate (in µM). Curves were fit using Prism 7. The presented Ki is the mean +/- s.e.m. of six independent experiments.

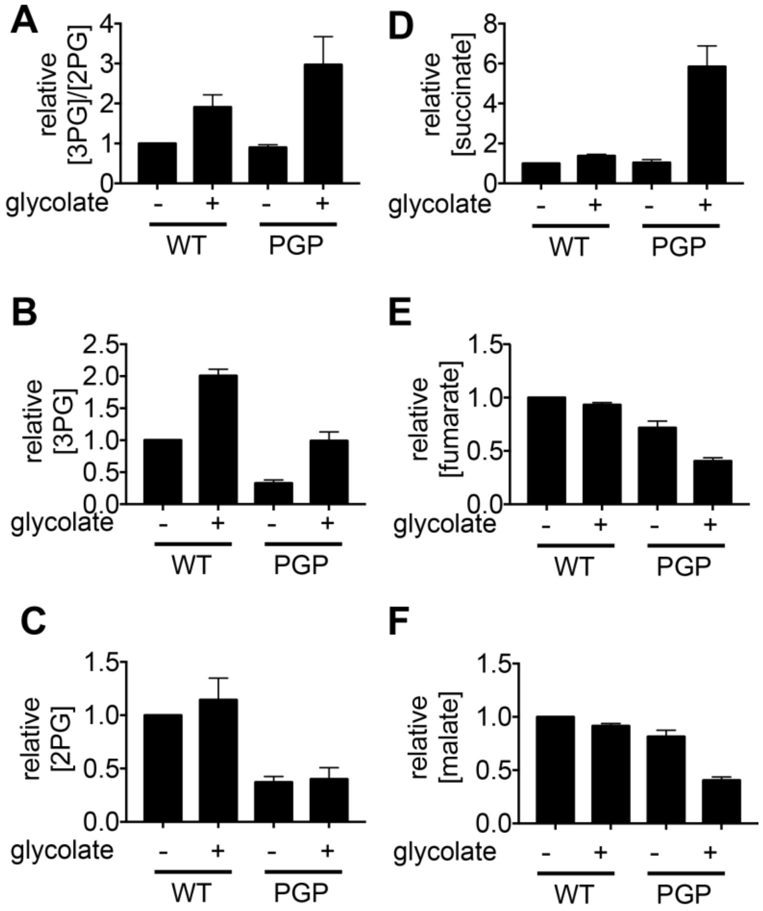

Supplementary Figure 7. Relative metabolite content in wild type and PGP knockout U2OS cells in the presence and absence of glycolate treatment.

The ratio of 3PG to 2PG (A), as well as 3-P-glycerate (3PG) (B), 2-P-glycerate (2PG) (C), succinate (D), fumarate (E) and malate (F) were measured by GC-MS and were normalized to the untreated wild type cells within each experiment. Values represent means ± s.e.m. of four independent experiments.

A

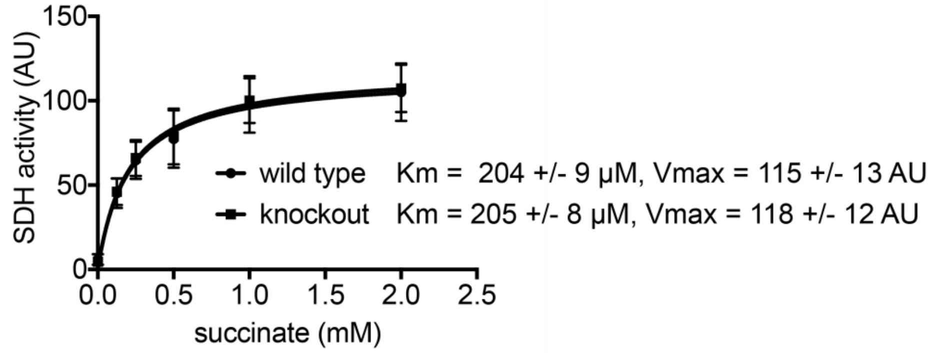

B

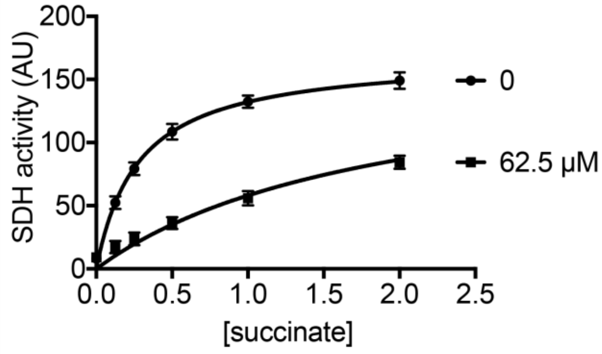

Supplementary Figure 8. Succinate dehydrogenase activity in extracts from HCT116 cells and its inhibtion by phosphoglycolate.
A, Succinate dehydrogenase (SDH) activity was determined in cellular extracts obtained from PGP wild type or knockout HCT116 cells. Estimates of Km and Vmax are means +/- s.e.m. (n=3).
B, Succinate dehydrogenase activity in PGP knockout HCT116 cells was determined in the presence or absence of 62.5 µM phosphoglycolate.
Presented values were normalized to protein concentrations and means +/- s.e.m. (n=3).

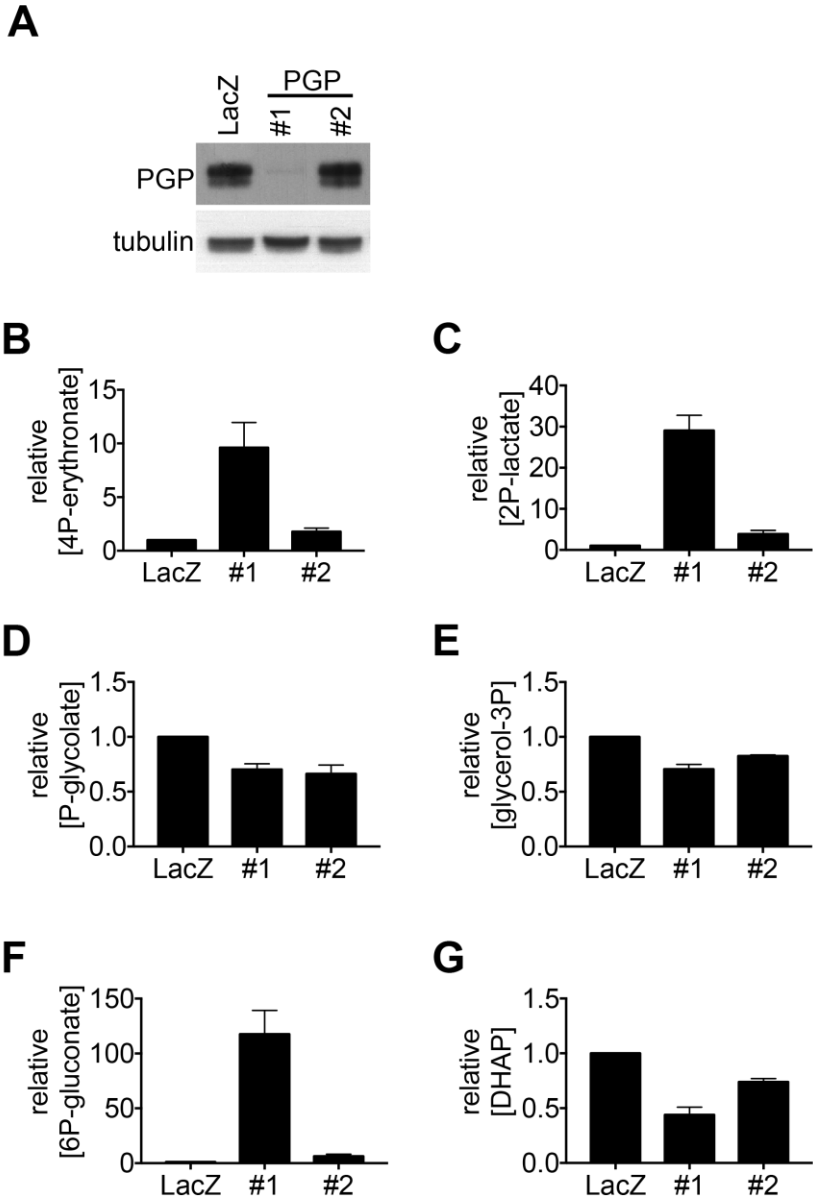

Supplementary figure 9: Metabolite changes in PGP-mutant human fibroblasts

A, Western blot analysis of immortalized human HFF2 fibroblasts upon lentiviral transduction with CRISPR/Cas9 constructs driving expression of a guide RNA targeting LacZ ("WT") or two guide RNAs targeting human PGP ("#1" and "#2"). As can be seen in this panel, polyclonal expression of guide-RNA #2 only leads to a minimal reduction of PGP levels, whereas guide RNA #1 leads to a knockout in almost all cells.

B-G, Metabolite concentrations were assessed by GC-MS in the indicated polyclonal cell lines and were normalized to the untreated wild type cells within each experiment. Values represent mean ± s.e.m. of four independent experiments.

Supplementary Table 1: Primers used in the current study

hGLYCTK_1	TGC TGT TGA CAG TGA GCG CCA CCC TGT TTC TTT CTG TGA ATA GTG AAG CCA CAG ATG TAT TCA CAG AAA GAA ACA GGG TGA TGC CTA CTG CCT CGG A
hGLYCTK_2	TGC TGT TGA CAG TGA GCG CGC CAT GCA AGG TGA TGT AAA ATA GTG AAG CCA CAG ATG TAT TTT ACA TCA CCT TGC ATG GCT TGC CTA CTG CCT CGG A
hPKM_1	TGC TGT TGA CAG TGA GCG CGC CTA CCT GTA TGT CAA TAA ATA GTG AAG CCA CAG ATG TAT TTA TTG ACA TAC AGG TAG GCT TGC CTA CTG CCT CGG A
hPKM_2	TGC TGT TGA CAG TGA GCG CCT GTC CAG TTC CTT TAG AAA ATA GTG AAG CCA CAG ATG TAT TTT CTA AAG GAA CTG GAC AGA TGC CTA CTG CCT CGG A
hPGK1_1	TGC TGT TGA CAG TGA GCG ATG CTG ACA AGT TTG ATG AGA ATA GTG AAG CCA CAG ATG TAT TCT CAT CAA ACT TGT CAG CAG TGC CTA CTG CCT CGG A
hPGK1_2	TGC TGT TGA CAG TGA GCG CTG GCA CTT CTC TGT TTG ATG ATA GTG AAG CCA CAG ATG TAT CAT CAA ACA GAG AAG TGC CAA TGC CTA CTG CCT CGG A
Q_mGLYCTK_s	CTG CGG GAA AAC CTC TAC CTA
Q_mGLYCTK_as	CGA TCC GGT AAG TTG TCC TCG
Q_U6_s	CGC TTC CGC AGC ACA TAT AC
Q_U6_as	TTC ACG AAT TTG CGT GTC AT
Q_PGK1_s	TGGACGTTAAAGGGAAGCGG
Q_PGK1_as	GCTCATAAGGACTACCGACTTGG
PGP_guide1_s	CAC CGG CAG CGG GCG TCG TCG CCA C
PGP_guide1_as	AAA CGT GGC GAC GAC GCC CGC TGC C
PGP_guide2_s	CAC CGG CGG CGC TCA GCC GCA CGC AG
PGP_guide2_as	AAA CCT GCG TGC GGC TGA GCG CCG CC

Supplementary Table 2. GC/MS analysis of metabolites

	Quantifyer m/z	Qualifyer m/z
**glycolate**	205 (206 for D)	
**phosphoglycolate**	357 (358 for D)	328
**2-P-lactate**	371	299
**4-P-erythronate**	357	561
**3-P-glycerate**	315	299, 387
**2-P-glycerate**	315	299, 387

**succinate**	247	129
**fumarate**	245	83
**malate**	233	335
**aspartate**	232	218
**PEP**	369	384
**DHAP**	315	299, 387
**glucose-6-P**	357	299, 315, 459
**6-P-gluconate**	387	315, 357
**fructose-6-P**	357	299, 315, 459
**glycerol-3-P**	357	387

References:

1 Parsons, J. L., Dianova, II and Dianov, G. L. (2004) APE1 is the major 3'-phosphoglycolate activity in human cell extracts. Nucleic acids research. **32**, 3531-3536

2 Xu, Y. J., Kim, E. Y. and Demple, B. (1998) Excision of C-4'-oxidized deoxyribose lesions from double-stranded DNA by human apurinic/apyrimidinic endonuclease (Ape1 protein) and DNA polymerase beta. J Biol Chem. **273**, 28837-28844

3 Inamdar, K. V., Pouliot, J. J., Zhou, T., Lees-Miller, S. P., Rasouli-Nia, A. and Povirk, L. F. (2002) Conversion of phosphoglycolate to phosphate termini on 3' overhangs of DNA double strand breaks by the human tyrosyl-DNA phosphodiesterase hTdp1. J Biol Chem. **277**, 27162-27168

4 Zhou, T., Akopiants, K., Mohapatra, S., Lin, P. S., Valerie, K., Ramsden, D. A., Lees-Miller, S. P. and Povirk, L. F. (2009) Tyrosyl-DNA phosphodiesterase and the repair of 3'-phosphoglycolate-terminated DNA double-strand breaks. DNA Repair (Amst). **8**, 901-911

5 Zhou, T., Lee, J. W., Tatavarthi, H., Lupski, J. R., Valerie, K. and Povirk, L. F. (2005) Deficiency in 3'-phosphoglycolate processing in human cells with a hereditary mutation in tyrosyl-DNA phosphodiesterase (TDP1). Nucleic acids research. **33**, 289-297

6 Collard, F., Baldin, F., Gerin, I., Bolsee, J., Noel, G., Graff, J., Veiga-da-Cunha, M., Stroobant, V., Vertommen, D., Houddane, A., Rider, M. H., Linster, C. L., Van Schaftingen, E. and Bommer, G. T. (2016) A conserved phosphatase destroys toxic glycolytic side products in mammals and yeast. Nature chemical biology. **12**, 601-607

7 Mugabo, Y., Zhao, S., Seifried, A., Gezzar, S., Al-Mass, A., Zhang, D., Lamontagne, J., Attane, C., Poursharifi, P., Iglesias, J., Joly, E., Peyot, M. L., Gohla, A., Madiraju, S. R. and Prentki, M. (2016) Identification of a mammalian glycerol-3-phosphate phosphatase: Role in metabolism and signaling in pancreatic beta-cells and hepatocytes. Proceedings of the National Academy of Sciences of the United States of America. **113**, E430-439

8 Segerer, G., Engelmann, D., Kaestner, A., Trotzmuller, M., Kofeler, H., Stigloher, C., Thiele, C., Jeanclos, E. and Gohla, A. (2018) A phosphoglycolate phosphatase/AUM-dependent link between triacylglycerol turnover and epidermal growth factor signaling. Biochimica et biophysica acta. **1863**, 584-594

9 Segerer, G., Hadamek, K., Zundler, M., Fekete, A., Seifried, A., Mueller, M. J., Koentgen, F., Gessler, M., Jeanclos, E. and Gohla, A. (2016) An essential developmental function for murine phosphoglycolate phosphatase in safeguarding cell proliferation. Scientific reports. **6**, 35160
